# QuickProt: A Fast and Accurate Homology‐Based Protein Annotation Tool for Non‐Model Organisms to Advance Comparative Genomics

**DOI:** 10.1111/1755-0998.70097

**Published:** 2026-01-12

**Authors:** Guisen Chen, Hehe Du, Zhenjie Cao, Ying Wu, Chen Zhang, Yongcan Zhou, Jingqun Ao, Yun Sun, Zihao Yuan

**Affiliations:** ^1^ Sanya Institute of Breeding and Multiplication, School of Marine Biology and Fisheries, Collaborative Innovation Center of Marine Science and Technology Hainan University Haikou Hainan China; ^2^ College of Marine Sciences, College of Life Sciences Fujian Agriculture and Forestry University Fuzhou China; ^3^ Engineering Research Center of Hainan Province for Blue Carbon and Coastal Wetland Conservation and Restoration Haikou China; ^4^ International Joint Research Center of Hainan Province for Blue Carbon and Coastal Wetland Haikou China; ^5^ CAS and Shandong Province Key Laboratory of Experimental Marine Biology, Institute of Oceanology, CAS Center for Ocean Mega‐Science Chinese Academy of Sciences Qingdao China

**Keywords:** comparative genomics, Epinephelinae, gene prediction, Rutaceae, *Xenopus*

## Abstract

The rapid growth of genome sequencing has outpaced the development of efficient annotation tools, especially for species lacking transcriptome data. To address this challenge, we present QuickProt, a fast, accurate and user‐friendly homology‐based protein annotation tool. QuickProt constructs a non‐redundant gene model by aligning homologous proteins from closely related species, offering an accurate and cost‐effective solution suitable for large‐scale comparative genomic studies. Benchmarking against BRAKER2 and GALBA across reference genomes demonstrated that QuickProt offers high specificity and dramatically improved runtime, while maintaining competitive annotation accuracy. To demonstrate its utility, we applied QuickProt to diverse genomes, including a non‐model teleost ( *Epinephelus bruneus* ), two tetraploid *Xenopus* species and 11 Rutaceae plants. Across these datasets, QuickProt supported robust phylogenetic reconstruction, identification of conserved orthologs and detection of biologically functional genes, pathways, and chromosomal evolution mechanisms, regardless of genome ploidy. Notably, it revealed a potential horizontal gene transfer event between groupers and *Vibrio*, and uncovered conserved modules involved in volatile oil biosynthesis and oil gland development in citrus. With its scalability and minimal computational demands, QuickProt provides a powerful platform for genome annotation and evolutionary inference. As the number of sequenced genomes continues to expand, QuickProt is a useful tool for accelerating comparative genomics and functional exploration across the tree of life.

## 1 Introduction

With the rapid advancement of genome sequencing technologies, the number of sequenced genomes has grown dramatically. However, according to the statistics from NCBI Genomes (as of March 12, 2025), only ~18% of eukaryotic genomes have completed gene model annotations. This substantial annotation gap remains a major obstacle in the advancement of comparative genomics, particularly in studies focused on gene structure, function and evolution.

One key bottleneck is the lack of efficient and broadly applicable strategies. As noted by the developers of GALBA (Brůna et al. 2023), major genome repositories (e.g., EBI, NCBI and Ensembl) face both computational and staffing limitations, preventing them from applying their internal annotation pipelines to all submitted genomes. Furthermore, existing annotation pipelines vary in performance across various taxa and often require substantial computational resources, making them impractical for smaller or less‐experienced research groups (Dimonaco et al. 2022). While RNA‐seq data can significantly enhance annotation accuracy, such data are usually unavailable due to sampling challenges or budgetary constraints.

In the absence of transcriptomic evidence, homology‐based protein annotation using splice alignment with proteins from related species remains a widely used strategy. Several tools have been developed for this purpose, such as blastp (Camacho et al. 2009), Spaln2 (Iwata and Gotoh 2012), GenomeThreader (Gremme et al. 2005), genewise (Birney et al. 2004), Exonerate (Slater and Birney 2005), genblastG (She et al. 2011), GeMoMa (Keilwagen et al. 2019) and Miniprot (Li 2023). However, these tools are either CPU‐intensive and can only align hundreds or thousands of proteins per CPU hour, limiting their practicality for medium to large genomes. Or, these tools align protein sequences individually, lacking the capacity to consolidate identical gene models or resolve conflicts when multiple proteins are mapped to the same genomic locus. Integrated pipelines like BRAKER3 (Gabriel et al. 2024) and MAKER2 (Holt and Yandell 2011) combine multiple sources of evidence (ab initio prediction, homology‐based protein alignment and transcriptome‐supported evidence integration), but they remain resource‐intensive and less practical when transcriptomic data are unavailable.

Some pipelines of homology annotation with self‐training frameworks, such as BRAKER2 (Brůna et al. 2021) and GALBA, attempted to address these limitations by iteratively refining ab initio models via GeneMark‐ES (Lomsadze et al. 2005) or AUGUSTUS (Stanke et al. 2006). However, these approaches had two main drawbacks: the increased computational costs and the risk of overfitting, which can compromise generalizability across diverse taxa (Hoff et al. 2019). For example, our test using BRAKER2 and GALBA on the long tooth grouper ( *Epinephelus bruneus* ) genome revealed that the predicted gene models were overly fragmented and exhibited high false‐positive rates. These complications had complicated the downstream comparative analysis by inflating gene counts or mischaracterizing gene family expansion.

Given that genome annotation remains a critical and often rate‐limiting step in phylogenomic studies (Dylus et al. 2024), there is an urgent need for tools that are both fast and accurate. QuickProt was developed to fill this gap. Unlike tools that require transcriptome data or complex model training, QuickProt employs a simplified three‐step pipeline to align homologous proteins, build pseudo‐transcripts and extract coding regions. By avoiding resource‐intensive steps while maintaining high precision, QuickProt significantly accelerates genome annotation, making it ideal for large‐scale or multiple‐species comparative genomics studies.

## 2 Materials and Methods

### 2.1 Genome Data Collection and Preprocessing

To evaluate the reliability and applicability of QuickProt, we collected the genomic and protein data from both animal (Epinephelinae and *Xenopus*) and plant (Rutaceae) species. For Epinephelinae, we obtained genome assemblies of *E. bruneus* and *E. moara*, along with protein sequences from seven related species ( *E. lanceolatus* , *E. fuscoguttatus* , *E. moara*, *E. akaara* , *Centropristis striata* , *Plectropomus leopardus* and *Cromileptes altivelis* ). These were retrieved from NCBI, Dryad, Figshare and NGDC databases. All *Xenopus* genomes and reference proteins were downloaded from NCBI. For Rutaceae, we downloaded genome assemblies and annotation files of 33 species from the NCBI and CPBD databases. Genomes of four model organisms were obtained from the Ensemble database and used for comparative benchmarking. To ensure non‐redundancy, all gene models were processed using the get_longest_transcript_gff3.py script (available in QuickProt), which retains only the longest isoform per gene. Detailed data sources are provided in Table S1.

### 2.2 Comparative Benchmarking of QuickProt, BRAKER2 and GALBA

To comprehensively benchmark QuickProt, we compared its performance against BRAKER2 and GALBA using the genome of *E. bruneus* , a species without corresponding transcriptome data. All pipelines were executed independently on Ubuntu 22.04 with 24 threads and default parameters. Runtime was measured using the Linux time command. The predicted gene models were evaluated using stat_gff3.py (available in QuickProt) to calculate metrics such as gene count, average coding sequences (CDS) number per gene, average CDS length, average CDS length per gene and the ratio of mono‐exonic to multi‐exonic genes.

For accuracy evaluation, we compared the predicted gene sets with curated annotations from four model organisms ( *Arabidopsis thaliana* , *Caenorhabditis elegans* , *Drosophila melanogaster* and *Takifugu rubripes* ). Sensitivity and specificity (Also known as precision) were calculated using GffCompare (v0.12.10) (Pertea and Pertea 2020). F1‐score was calculated as follows (Brůna et al. 2023): F1‐score = (2 × Sensitivity × Specificity)/(Sensitivity + Specificity). To avoid resource conflict, each tool was executed independently.

### 2.3 Function Annotation and Repetitive Element Analysis

To explore the biological relevance of a predicted gene model, we performed functional annotation by aligning the predicted protein‐coding genes to the Non‐Redundant (NR) database, Kyoto Encyclopedia of Genes and Genomes (KEGG) and SwissProt using DIAMOND (v2.1.8.162) (Buchfink et al. 2015), with an *e*‐value cutoff of 1e‐10. The top hit was retained as the final annotation for each gene. To determine whether predicted genes reside in transposable elements (TE), particularly those containing reverse transcriptase domains, we combined de novo and homology‐based prediction methods to identify genomic repetitive elements. For the de novo prediction, we used RepeatModeler (v2.0.5), which integrates results from recon (v1.08) and repeatscout (v1.0.6). Unclassified repeats were annotated using DeepTE. We also used the sequence library from LTR_retriever (v2.9.9), which integrates predictions from LTR_FINDER_parallel (v1.1) and genometools (v1.6.2). Homology‐based predictions were conducted using RepeatMasker (v4.1.6), incorporating both de novo libraries and the Epinephelinae subsets from Dfam (v38). The RepeatMasker was run twice to reduce misclassification of unknown TEs.

### 2.4 Completeness Assessment of Gene Predictions

To assess gene models' completeness, we conducted BUSCO (Benchmarking universal single‐copy orthologs) assessments via compleasm (v0.2.6) (Huang and Li 2023), using the actinopterygii_odb10 lineage dataset. Proteins predicted from QuickProt, BRAKER2, GALBA and seven closely related species ( *E. lanceolatus* , *E. fuscoguttatus* , *E. moara*, *E. akaara* , *C. striata* , *P. leopardus* and *C. altivelis* ) were included in the analysis.

### 2.5 Phylogenetic and Comparative Genomics in Epinephelinae

To explore the phylogenetic relationships, protein‐coding genes from eight species ( *E. lanceolatus* , *E. fuscoguttatus* , *E. moara*, *E. akaara* , *C. striata* , *P. leopardus* , *C. altivelis* and *E. bruneus* ) were aligned using DIAMOND (v2.1.8.162). Orthologous gene families were identified using OrthoFinder (v2.5.5) (Emms and Kelly 2019). Single‐copy genes were aligned using MAFFT (v7.525) (Katoh and Standley 2013), and a phylogenetic tree was constructed with FastTree (Price et al. 2009) using maximum likelihood. The species tree was rooted using the MSA (multiple sequence alignment) method (Emms and Kelly 2018). Synteny between *E. moara* and *E. bruneus* was assessed using JCVI (v1.4.23) (Tang et al. 2024), with a c‐score threshold set to 0.99 and a minspan of 30.

To infer the horizontal gene transfer (HGT), relative synonymous codon usage (RSCU), and GC content were calculated using pyCUBs (https://github.com/thecgs/pyCUBs). Genomes included in the analysis were *V. parahaemolyticus* (GCF_000196095.1), *E. bruneus* , *P. leopardus* and the HGT candidate clade.

### 2.6 Identification and Phylogenetic Analysis of Reverse Transcriptase Domain‐Containing Proteins

Reverse transcriptase domain‐containing proteins from *E. bruneus* were used to search genomes of Amphibia, Cyclostomata, Osteichthyes and Reptilia using genblastG (v1.0.138) (Table S1), with the parameters ‘‐e 1e‐10 ‐r 5 ‐c 0.8’. Domains were predicted using hmmscan (v3.4) (Finn et al. 2011) in the Pfam database (v36.0), with *e*‐value set to 1e‐5. Candidates containing the RVT_1 domain (Table S2) were aligned with MAFFT (v7.525), and the phylogenetic analysis was conducted via IQ‐TREE2 (v2.3.5) (Minh et al. 2020), with 1000 bootstrap replicates, and the best model (VT + F + G4) was selected based on BIC.

### 2.7 Gene Prediction, Phylogenetic and Synteny Analysis of *Xenopus*

To validate the applicability of QuickProt in polyploid organisms, we used protein sequences from *Xenopus laevis* and *X. tropicalis* as reference inputs to predict gene models for *X. petersii* and *X. borealis* , respectively. The construction of the species tree follows the same procedure as for Epinephelinae. Comparative analysis of synteny among subgenomes was performed using JCVI (v1.4.23).

### 2.8 QuickProt Annotation in 11 Rutaceae Species

To further evaluate the applicability of QuickProt, we annotated protein‐coding genes in 11 Rutaceae species with no predicted protein annotations. Protein sequences from 22 Rutaceae species were pooled and used as input (Table S1). For more distantly related species ( *M. koenigii* and *C. heptaphylla*), the identity threshold was lowered to 0.8 to avoid underestimation. All other parameters remained as the default.

### 2.9 Phylogenetic Analysis and Divergence Time Estimation in Rutaceae

Phylogenetic and ortholog inference in Rutacea followed the same approach used for Epinephelinae. Codon alignments were generated using PAL2NAL (v14.1) (Suyama et al. 2006), and divergence times were estimated using the mcmctree model within the PAML (v4.10.7) (Yang 2007), using the correlated rate clock model (clock = 3). Calibration points included the split between *Citrus* and *Poncirus trifoliata* , which was constrained to 7–10 Mya (Yin et al. 2025). Parameters were set as follows: alpha gamma = G (1, 1), kappa gamma = G (6, 2), rgene gamma = G (2, 20), sigma2 gamma = G (1, 10), respectively. After the first 40,000,000 iterations were discarded as burn‐in, the MCMC ran sampling every 100 iterations until 1,000,000 samples were reached. The effective sample size (ESS) for each node age and parameter was determined in Tracer (v1.72), and ESS values were verified to exceed 200.

### 2.10 Core Genes Identification and Functional Enrichment in the Rutaceae

The longest representative gene model within each family is selected for downstream analysis. Functional annotations were conducted using DIAMOND (v2.1.8.162) against NR, KEGG and SwissProt databases with an *e*‐value threshold of 1e‐10. Core gene families were defined as those present in all Rutaceae species. Functional enrichment analysis was conducted via clusterProfiler (Wu et al. 2021) with *p*‐value < 0.05.

## 3 Results

### 3.1 QuickProt Algorithm Overview

**FIGURE 1:**
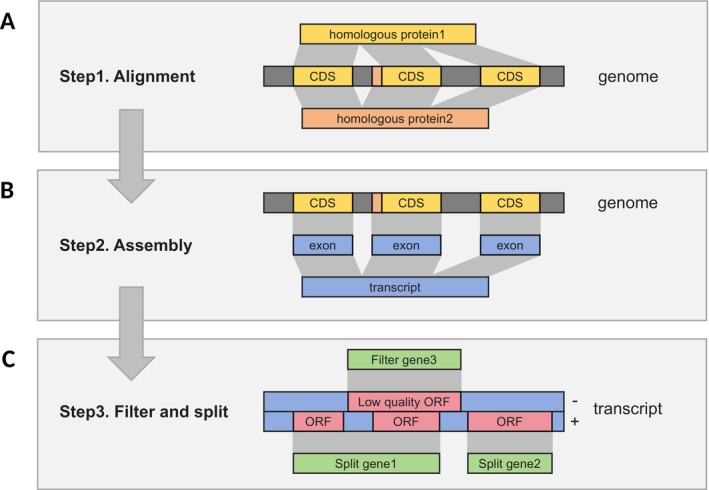
Schema of the QuickProt algorithm. (A) This step uses Miniprot to align protein sequences to the genome. (B) This step delineates coding regions and assembles them into pseudo‐transcripts. (C) This step predicts gene structures within the pseudo‐transcripts, splits overlapping genes based on their positional information and filters out low‐quality genes according to their length and prediction scores.

The QuickProt algorithm operates in three major steps (Figure 1). Firstly, it utilises miniprot (v0.12) to align homologous protein sequences with the target genome. These aligned regions are expanded and assembled into pseudo‐transcripts, although they lack the untranslated regions (UTRs). Next, TransDecoder (v5.7.1) (Haas et al. 2013) is used to predict the coding regions within these pseudo‐transcripts. Finally, low‐quality gene models are filtered out based on sequence length and score. In rare cases, where a pseudo‐transcript contains sequences from multiple transcripts due to overlapping genes, QuickProt resolves these conflicts by splitting the pseudo‐transcript based on open reading frame (ORF) prediction scores and sequence length. This ensures the construction of accurate and non‐redundant gene models, which are suitable for downstream analysis.

In essence, QuickProt functions similarly to the blotting method. It predicts gene models by ‘tagging’ the conserved coding regions using the homologous proteins from closely related species. This approach is highly efficient, minimising computational costs while maintaining annotation accuracy. A key limitation of this approach is its reliance on high‐quality homologous input sequences, as it cannot predict genes lacking homology support. To mitigate this, we recommend using comprehensive and well‐curated reference protein datasets, such as those available from the Orthologous Database for Proteins (OrthoDB; https://bioinf.uni‐greifswald.de/bioinf/partitioned_odb12). Alternatively, retrieving protein sequences from closely related species within the same genus from NCBI is also a practical solution. Nevertheless, with the continuous expansion of reference protein databases (e.g., SwissProt, OrthoDB), this limitation is expected to become progressively less significant.

### 3.2 Performance of QuickProt on the *E. brueus* Genome

**FIGURE 2:**
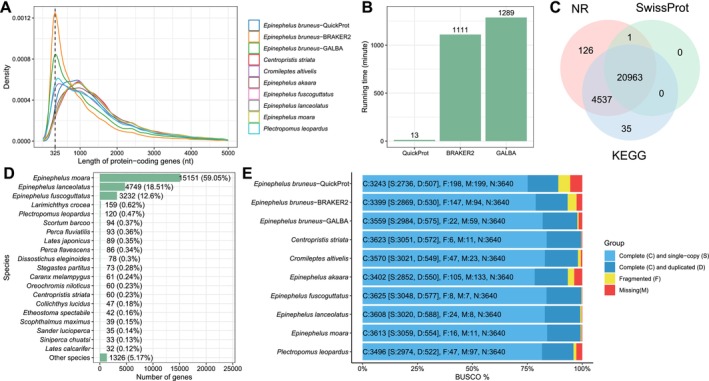
Performance and annotation overview of QuickProt in teleost genomes. (A) Density distribution of protein‐coding gene length. (B) Comparison of processing time for QuickProt, BRAKER2 and GALBA using 24 threads on Ubuntu 22.04. (C). Venn diagram showing the overlap of the annotation categories of *E. bruneus* protein‐coding genes annotated by QuickProt. (D) Distribution of best‐hit species from NR database annotation using QuickProt predicted protein coding genes from *E. bruneus* . (E) BUSCO evaluation showing the genome completeness.

To evaluate the performance of QuickProt on a complex non‐model genome, we applied it to *E. bruneus* , a teleost lacking a high‐quality reference annotation. QuickProt predicted 26,046 protein‐coding genes, while BRAKER2 and GALBA predicted 55,940 and 39,395 genes, respectively. These higher counts from BRAKER2 and GALBA indicate substantially overprediction, as the expected gene number in six other closely related grouper species ranges from 23,722 to 25,965 genes (Table S3). To further assess the quality of gene models, we calculated the ratio of mono‐exonic genes to multi‐exonic genes. BRAKER2 and GABLA displayed a strong bias towards mono‐exonic predictions, suggesting fragmentation. In contrast, the gene model predicted by QuickProt is more consistent with other groupers in terms of average CDS number per gene, average CDS length, and average CDS length per gene. We also analysed the distribution of predicted gene lengths compared with seven closely related teleost species (Figure 2A). The protein‐coding genes predicted by BRAKER2 and GALBA showed a significant peak around 325 nt, indicating many short or fragmented predictions. In contrast, the gene length distribution from QuickProt is more closely mirrored by the natural distribution observed in reference species, suggesting a much lower rate of fragmented gene predictions. In terms of computational efficiency, QuickProt remarkably outperformed both BRAKER2 and GALBA, 1098 min faster than BRAKER2 and 1276 min faster than GALBA (Figure 2B). This improvement is largely because QuickProt avoids computationally extensive de novo training steps. In addition, to assess the annotation accuracy, we searched the predicted proteins against NR, KEGG and SwissProt via DIAMOND. Of the 25,965 protein‐coding genes predicted by QuickProt, 20,963 (80.64%) had hits in all three databases and 25,662 (98.72%) had hits at least one database (Figure 2C). Most hits corresponded to known grouper proteins, including *E. moara* (59.05%), *E. lanceolatus* (18.51%) and *E. fuscoguttatus* (12.60%) (Figure 2D). Additionally, BUSCO analysis showed that QuickProt achieved 89.09% completeness, which although slightly lower than that of BRAKER2 (93.38%) and GALBA (97.77%), still reflects high‐quality annotation while maintaining extraordinary speed (Figure 2E).

Overall, these results illustrate QuickProt's robust capacity for accurate, efficient and biologically consistent gene prediction in non‐model genomes, especially those with limited transcriptomic data.

### 3.3 Benchmarking of QuickProt on Model Organisms

**FIGURE 3:**
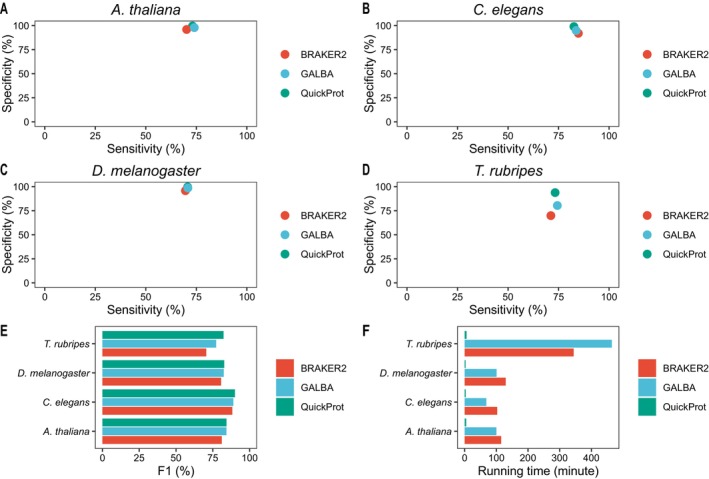
Benchmarking of QuickProt against BRAKER2 and GALBA across four model organisms. (A–D) Sensitivity and specificity of gene prediction at the base level in four reference genomes. (E) Comparison of F1‐score for gene prediction across four genomes. (F) Running time of BRAKER2, GALBA and QuickProt in each genome, demonstrating computational efficiency.

To validate QuickProt's general applicability, we conducted benchmark tests on four model organisms, *A. thaliana* , *C. elegans* , *D. melanogaster* and *T. rubripes* . Across all species, QuickProt achieved the highest specificity among the tested tools (Figure 3A–D). This can be attributed to its design, which only detects regions that are highly homologous to the reference sequence, thereby reducing false positives and overall runtime. In terms of sensitivity, QuickProt performed between GALBA and BRAKER2, and its predicted gene numbers were correspondingly lower. Nevertheless, the method maintained a robust balance between precision and recall (Table S4). The *F*‐score of BRAKER2, GALBA and QuickProt reflects the overall annotation accuracy. The results showed that QuickProt's accuracy is comparable to that of existing mainstream pipelines (Figure 3E). Notably, QuickProt's most significant advantage is its runtime performance. By eliminating the need for de novo model training, QuickProt extensively reduces the processing time (Figure 3F), making it particularly well‐suited for large‐scale comparative genomics and rapid annotation of those newly sequenced species.

### 3.4 Case Study 1: Comparative Genomics of the Epinephelinae

**FIGURE 4:**
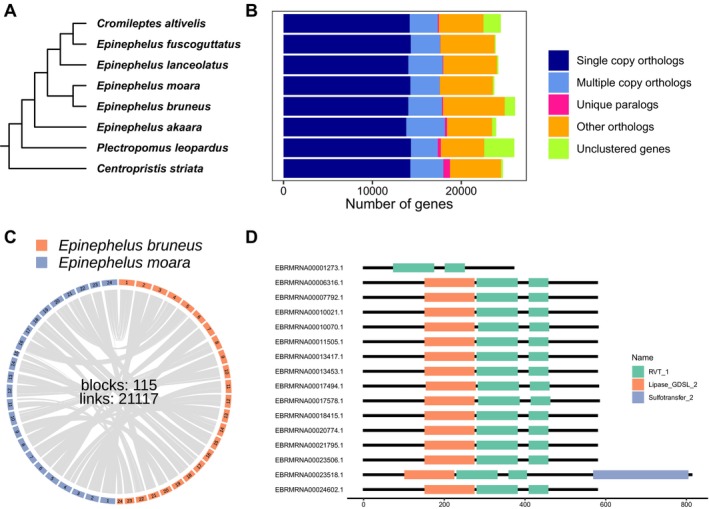
Comparative genomics and protein domain analysis in groupers. (A) The phylogenetic tree of groupers with *C. striata* as the outgroup. (B) Number of orthologous genes identified between groupers and *C. striata*. (C) Genome synteny between *E. moara* and *E. bruneus* . (D) Domain structure of reverse transcriptase domain‐containing proteins in *E. bruneus* .

To demonstrate QuickProt's utility in comparative and evolutionary genomics, we applied it to the Epinephelinae family to explore its phylogenetic relationship and evolutionary paths. Using protein‐coding genes annotated by QuickProt, *E. bruneus* showed the highest sequence homology (59.05%) with *E. moara*, consistent with prior morphological and taxonomic evidence (Guo et al. 2014, 2009). To further explore the relationship among grouper species, we reconstructed the phylogenetic tree of the Epinephelinae from 12,470 single‐copy orthologs. As expected, *E. bruneus* and *E. moara* clustered into the same clade, while *P. leopardus* occupied the basal position within groupers, and *C. striata* served as an outgroup (Figure 4A). The phylogenetic topology is fully consistent with earlier studies based on nuclear and mitochondrial genomes (Cao et al. 2022; Xie et al. 2022; Yang et al. 2021; Zhuang et al. 2013). Moreover, gene family analyses revealed highly similar distributions among grouper species (Figure 4B, Table S5). Further comparative genomic analysis revealed strong synteny: 21,117 collinear genes and 115 collinear blocks were identified between *E. bruneus* and *E. moara*. The high‐level genomic collinearity between these species underscores their genomic consistency (Figure 4C).

**FIGURE 5:**
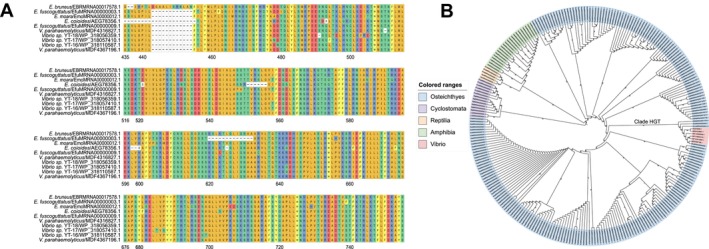
Evolutionary analysis of reverse transcriptase domain‐containing proteins. (A) Alignment of proteins in the clade inferred to be derived from horizontal gene transfer (HGT). (B) Phylogenetic tree of the reverse transcriptase domain‐containing proteins across species.

**FIGURE 6:**
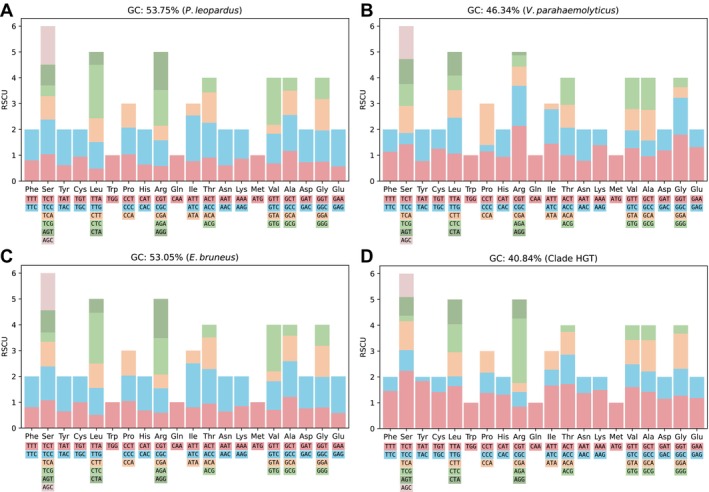
Comparative analysis of relative synonymous codon usage (RSCU). (A) RSCU pattern in *P. leopardus* . (B) RSCU in *V. parahaemolyticus* . (C) RSCU in *E. bruneus* . (D) RSCU in the HGT derived clade.

Interestingly, beyond comparative genomics, QuickProt also facilitated the discovery of a potential HGT event in *E. bruneus*. A cluster of proteins containing the RVT_1 domain was identified in the *E. bruneus* (Figure 4D). These proteins exhibited high similarity to reverse transcriptase domain‐containing proteins from *Vibrio* and an uncharacterized protein in *E. lanceolatus* (Figure 5A). These proteins were embedded within LINE/L2 transposons (Table S6), suggesting a possible transposon‐mediated HGT event. In addition, phylogenetic analysis further supports this inference, as the *Vibrio* sequences clustered within the grouper clade (Figure 5B). Furthermore, RSCU and GC content analysis revealed similar codon preferences between *Vibrio* and the HGT gene cluster (e.g., TTT over TTC for Phe; TCT over TGC for Cys, AGA over AGG for Arg) (Figure 6). Collectively, these results demonstrate QuickProt's strength not only in precise genome annotation but also in enabling downstream comparative and evolutionary analyses, from resolving phylogenetic relationships to uncovering adaptive genomic innovations such as HGT potentially shaped by long‐term host–pathogen interactions in marine environments.

### 3.5 Case Study 2: Application of QuickProt in Polyploid Species

To assess the performance of QuickProt in polyploid genomes, we applied it to two *Xenopus* species. In *X. petersii* , QuickProt, BRAKER2 and GALBA predicted 37,705, 102,771 and 68,119 genes, respectively, while in *X. borealis*, these tools predicted 24,506, 68,788 and 47,189 genes. The substantially inflated gene counts produced by BRAKER2 and GALBA indicate a strong tendency to overestimate the number of genes (Table S3).

**FIGURE 7:**
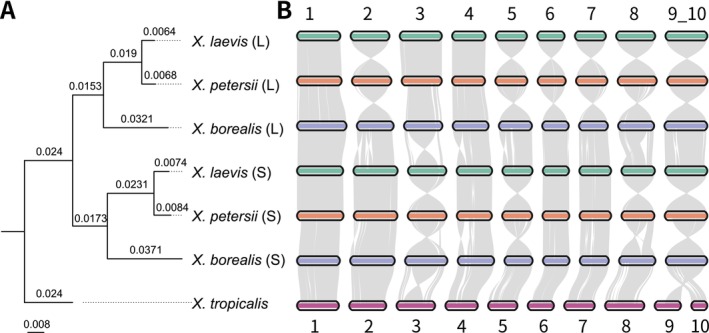
Comparative genomics analysis in *Xenopus*. (A) Phylogenetic tree based on protein‐coding genes of *Xenopus*. The suffixes ‘L’ and ‘S’ used after the species names denote large and small subgenomes, respectively. (B) Synteny analysis in *Xenopus* species.

To elucidate the evolutionary relationships among tetraploid *Xenopus* species, we reconstructed a phylogeny comprising the large (L) and small (S) subgenomes of the tetraploid species *X. laevis* , *X. petersii* and *X. borealis* , along with the diploid species *X. tropicalis* (Figure 7A). Consistent with previous phylogenetic studies (Evans et al. 2024), *X. laevis* was found to be more closely related to *X. petersii* than to *X. borealis* . The phylogeny also revealed that the L and S subgenomes of all three tetraploid species ( *X. laevis* , *X. petersii* and *X. borealis* ) each formed distinct, separate clades, supporting the view that these species originated from allopolyploidization events involving two distinct ancestral lineages. Additional genomic synteny analysis further clarified the chromosomal evolution of *Xenopus*. In all tetraploid species, chromosomes 9_10 appeared to have arisen through the fusion of chromosomes 9 and 10 found in the diploid ancestor. Moreover, the two subgenomes display marked asymmetry: the S subgenome has undergone more extensive gene loss (including pseudogenization and deletions) and intrachromosomal rearrangements, whereas the L subgenome has retained a more intact ancestral genomic structure (Figure 7B). These findings are consistent with previous research (Session et al. 2016).

Together, these results demonstrate that QuickProt can effectively annotate genes across complex polyploid genomes and accurately reconstruct subgenome‐specific evolutionary paths. Its application to *Xenopus* highlights its potential for revealing patterns of genome evolution and chromosomal rearrangement in non‐model polyploid organisms.

### 3.6 Case Study 3: Comparative Genomics of Rutaceae

To assess QuickProt's scalability and utility in large‐scale comparative genomics, we applied it to annotate protein‐coding genes in 11 Rueaceae species. The predicted gene counts ranged from 23,303 to 41,083, which is consistent with previous estimates and supports the accuracy of QuickProt (Guardo et al. 2021; Nakandala et al. 2024; Yang et al. 2023). Among the predicted 11 species, the average CDS number per gene, average CDS length, average CDS length per gene and the ratios of mono‐exonic to multi‐exonic genes range from 3.82–4.75, 240.66–282.85 nt, 962.87–1182.81 nt and 0.2–0.29, respectively. These results were comparable with the statistics from 22 other Rutaceae genomes (Table S3), highlighting QuickProt's utility in standardising annotation across large datasets.

**FIGURE 8:**
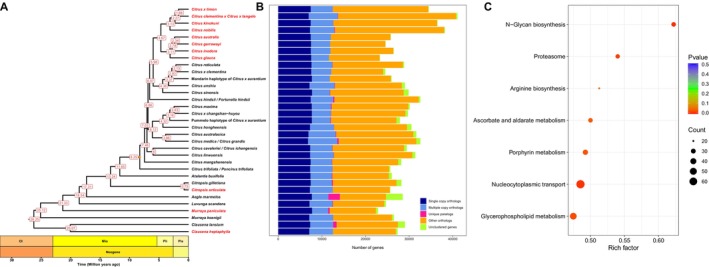
Comparative genomics and functional enrichment in the Rutaceae family. (A) Phylogenetic tree and estimated divergence time among Rutaceae species (Species names in red are annotated by QuickProt). (B) Number of orthologous gene groups shared within the Rutaceae species. (C) KEGG enrichment of the core genes across the Rutaceae family.

Among the 33 Rutaceae species analysed, 97.9% of predicted genes were assigned to 36,884 orthogroups. A phylogenetic tree constructed from 2510 single‐copy orthologs revealed the evolutionary relationships among these species (Figure 8A). Notably, the two haplotypes of the hybrid species *C. aurantium* clustered with *C. maxima* and *C. reticulata* , which is consistent with the previous report that *C. aurantium* is an F1 hybrid of pure *C. maxima* and *C. reticulata* parents (Wu et al. 2014). Among hybrid cultivars, *C. limon* and *C. clementina × C. tangelo* were placed at the top of the phylogenetic tree, with the placement of *C. limon* aligning well with previous reports (Wang et al. 2017). Although the phylogeny of *C. clementina × C. tangelo* has not been well documented, its position in our analysis appears biologically plausible. Additionally, QuickProt‐based annotations confirmed that *C. articulata* , *M. paniculata* and *C. heptaphylla* were accurately clustered with their respective genera (Figure 8A), further supporting the robustness and broad applicability of QuickProt in large‐scale phylogenetic studies.

In addition to phylogenetic topology, the divergence time estimates showed that the Australian citrus lineage diverged during the early Pliocene (around 4.67 Mya), which is in strong agreement with previous research (Wu et al. 2018). Functional analysis revealed 9169 conserved gene families (408,157 genes) across Rutaceae (Figure 8B and Table S7), which were enriched in pathways such as proteasome function, N‐Glycan biosynthesis and lipid metabolism (Figure 8C, Table S8). Further analysis of core genes highlighted their predominant roles in transcriptional regulation, stress responses and key metabolic processes, underscoring their essential functions in development and adaptation (Table S9). Within the glycerophospholipid metabolism pathway, we identified 47 candidate genes (Table S10), including *DGK*, *GDPD*, *GPAT* and *LPAT2*, many of which are known to play key roles in lipid signalling and oil accumulation (Bai et al. 2021; Kue Foka et al. 2020; Liao et al. 2025; Yin et al. 2020). Additionally, QuickProt also enabled the identification of transcriptional regulators associated with oil gland development, such as *LMI1*, *DRNL* and *MYC5*. These genes are crucial for the biosynthesis of volatile oils, the hallmark traits of the Rutaceae family (Wang et al. 2024).

Overall, our results demonstrate that QuickProt provides high reliability and biological relevance in protein annotation across both model and non‐model organisms. Its scalability and precision make it a powerful tool for phylogenetic and functional analysis in comparative genomics.

## 4 Discussion

In this study, we present QuickProt, a novel homology‐based annotation tool designed for rapid, accurate and low‐cost prediction of protein‐coding genes, particularly in non‐model organisms. Benchmarking results using the *E. bruneus* genome and four model species demonstrated that QuickProt not only outperformed BRAKER2 and GALBA in computational efficiency but also exhibited superior control over false positives, resulting in more reliable gene model predictions. In addition, compared to other tools, QuickProt shows a reduced tendency to over‐predict mono‐exonic genes, and the CDS length distribution generated by QuickProt more closely reflects the natural patterns observed in related species.

Despite its strengths, QuickProt does have certain limitations that should be acknowledged. It relies on TransDecoder to identify candidate coding regions. Since TransDecoder heavily relies on the input sequences to train species‐specific models, this may cause limited transcript assembly in QuickProt, especially in cases where only a few homologues are available (Haas et al. 2013). The recent release of TD2, which employs a pre‐trained protein model capable of analysing small sets or even individual transcripts (Mao et al. 2025), offers a solution to this issue. The forthcoming QuickProt release will incorporate TD2 to mitigate this limitation. Moreover, QuickProt cannot identify novel genes lacking protein homology, as it does not include a de novo prediction component. This may result in the omission of species‐specific genes. Therefore, for projects aiming to generate a comprehensive protein‐coding gene set, GALBA and BRAKER2 remain suitable options, despite substantially higher false‐positive rates and significantly higher computational demands (Brůna et al. 2021, 2023; Gabriel et al. 2024).

As with all annotation tools, there is no universal gene prediction solution or tool suitable for every species (Dimonaco et al. 2022). Nonetheless, QuickProt fills an important niche by offering a streamlined and broadly applicable solution when transcriptomes are unavailable, making it ideal for comparative genomics in under‐characterised lineages or early‐stage genome projects. Although QuickProt may be slightly less sensitive than fully integrated pipelines, it offers far greater speed, precision and ease of deployment, requiring minimal computational resources. These strengths make QuickProt particularly valuable for large comparative studies that demand consistent annotation across diverse taxa. As large‐scale sequencing efforts such as the Earth BioGenome Project continue to expand, the growing availability of homologous protein data from related species will further enhance the utility and effectiveness of QuickProt (Dylus et al. 2024; Lewin et al. 2018).

In our application cases, QuickProt enabled annotations and phylogenetic reconstruction of both Animal (Epinephelinae, *Xenopus*) and Plant (Rutaceae) species, regardless of their ploidy levels. The resulting phylogenies were highly consistent with previous studies (Guardo et al. 2021; Guo et al. 2014; Wu et al. 2014, 2018; Zhuang et al. 2013; Evans et al. 2024), validating the reliability and broad applicability of QuickProt for robust phylogenetic inference across diverse taxa. Remarkably, our analysis in groupers uncovered a potential HGT event from *Vibrio*, likely mediated by LINE/L2 transposable elements. This inference was supported by codon usage bias and domain structure analysis (Sun et al. 2015). Such findings illustrate QuickProt's utility not only for genome annotation but also as a foundation for discovering novel evolutionary mechanisms in eukaryotes, such as HGT, which has also been reported mainly in plants (Yang et al. 2019), insects (Gasmi et al. 2021), and fish (Graham and Davies 2021; Sun et al. 2015). Given that *Vibrio* is a main pathogen in grouper aquaculture (Ybañez Jr and Gonzales 2023), its long‐term infection pressure may have promoted HGT events (Xu et al. 2024). This may potentially facilitate the evolution of immune genes; these observations revealed the potential application of QuickProt as a powerful tool for exploring evolutionary mechanisms from genomic resources with greater efficiency. Moreover, QuickProt exhibited strong performance in annotating polyploid genomes, which are often challenging due to their complexity and frequent genomic rearrangements. In *Xenopus* species, QuickProt successfully resolved subgenomic structures and detected genomic rearrangements consistent with previous reports, providing further evidence that the S subgenome is undergoing more active genomic changes than its L counterpart. These results broaden our understanding of genome evolution in polyploid species and underscore the potential of QuickProt for investigating genome formation and evolutionary dynamics in non‐model organisms.

In plants, QuickProt enabled the identification of core gene families involved in volatile oil biosynthesis and oil gland development across Rutaceae species. Core genes such as *LPAT2*, *MYC5*, *LMI1* and *GPAT* were identified to be involved in lipid metabolism and stress resistance (Bai et al. 2021; Wang et al. 2024; Yin et al. 2020). Those genes emerged as promising targets for functional validation or genetic improvement in economically important crops like citrus.

In conclusion, QuickProt offers a streamlined, accurate and efficient solution to meet the pressing need for effective genome annotations. We tested its performance across both model and non‐model organisms, highlighting its versatility and robustness. As the number of sequenced genomes continues to increase, QuickProt is expected to have an increasingly important role in accelerating genomic discovery across the tree of life, particularly for areas requiring rapid, consistent and biologically relevant gene annotations.

## Author Contributions

Conceptualization: Yun Sun, Zihao Yuan and Guisen Chen; Software, formal analysis, validation and visualisation: Guisen Chen; Supervision: Hehe Du, Zhenjie Cao, Ying Wu, Chen Zhang, Yongcan Zhou, Jingqun Ao; Resources: Yongcan Zhou; Funding acquisition: Yun Sun. All authors contributed to the article and approved the submitted version. All authors have read and agreed to the published version of the manuscript.

## Conflicts of Interest

The authors declare no conflicts of interest.

## Supporting information


**Tables S1‐S10:** men70097‐sup‐0001‐Tables.xlsx.

## Data Availability

The QuickProt is implemented in Python and licensed under the MIT Licence. The source code, documentation and tutorials are available at https://github.com/thecgs/quickprot. In addition, we provide a singularity image file of QuickProt and the result files of comparative genomics analysis of Epinephelinae and Rutaceae (including the gene models predicted by QuickProt, functional annotations, species trees, etc.), which are archived on Zenodo (https://doi.org/10.5281/zenodo.15086699).
